# Association between CYP metabolizer phenotypes and selective serotonin reuptake inhibitors induced weight gain: a retrospective cohort study

**DOI:** 10.1186/s12916-022-02433-x

**Published:** 2022-07-26

**Authors:** Maria L. Ricardo-Silgado, Sneha Singh, Lizeth Cifuentes, Paul A. Decker, Daniel Gonzalez-Izundegui, Ann M. Moyer, Maria D. Hurtado, Michael Camilleri, Suzette J. Bielinski, Andres Acosta

**Affiliations:** 1 Precision Medicine for Obesity Program and Division of Gastroenterology and Hepatology, Department of Medicine, Mayo Clinic, Rochester, MN USA; 2 Division of Epidemiology, Department of Quantitative Health Research, Mayo Clinic, Rochester, MN USA; 3 Department of Laboratory Medicine and Pathology, Mayo Clinic College of Medicine and Science, Mayo Clinic, Rochester, MN USA; 4 Division of Endocrinology, Diabetes, Metabolism, and Nutrition, Department of Medicine, Mayo Clinic Health System, La Crosse, WI USA

**Keywords:** Pharmacogenomics, Weight gain, CYP metabolizer phenotypes

## Abstract

**Background:**

Prescription medications such as selective serotonin reuptake inhibitors (SSRIs), commonly used to treat depression, are associated with weight gain. The role of pharmacogenomics in predicting SSRI-induced weight gain is unclear.

**Methods:**

In this retrospective cohort study from participants in the Mayo Clinic RIGHT study who were prescribed citalopram, paroxetine, sertraline, or fluoxetine, our aim was to evaluate the association of metabolizer phenotype and total body weight after 6 months of SSRIs initiation. We evaluated the metabolizer phenotypes (poor/intermediate, normal, and rapid/ultra-rapid) of the cytochromes P450 enzymes genes: *CYP2C9, CYP2C19,* and *CYP2D6* known to influence the metabolism of SSRI medications: *CYP2C19* for citalopram, *CYP2D6* for paroxetine, *CYP2D6* and *CYP2C19* for sertraline, and *CYP2D6* and *CYP2C9* fluoxetine. In addition, we assessed the association of metabolizer phenotype and total body weight change at six months following SSRI prescription using parametric analysis of covariance adjusted for baseline body weight and multivariate regression models.

**Results:**

*CYP2C19* poor/intermediate metabolizers prescribed citalopram gained significantly more weight than normal or rapid/ultra-rapid metabolizers at 6 months (TBWG %: 2.6 [95% CI 1.3—4.1] vs. 0.4 [95% CI -0.5 – 1.3] vs. -0.1 [-95% CI -1.5—1.1]; *p* = 0.001). No significant differences in weight outcomes at six months of treatment with paroxetine, sertraline, or fluoxetine were observed by metabolizer status.

**Conclusions:**

Weight gain observed with citalopram may be mediated by *CYP2C19* metabolizer status.

**Supplementary Information:**

The online version contains supplementary material available at 10.1186/s12916-022-02433-x.

## Background

Obesity is a chronic and complex multifactorial disease associated with multiple metabolic comorbidities, such as type 2 diabetes mellitus, and psychiatric diagnoses such as major depressive and anxiety disorders. There is a bidirectional relation between depression and obesity; thus, patients with obesity have a 55% increased risk of being diagnosed with depression over time, and patients with depression have a 58% increased risk of developing obesity [1].

Selective serotonin reuptake inhibitors (SSRIs) are the first-line treatment for depression. One side effect of their use is body weight gain during short- and long-term management. In fact, it is reported that patients prescribed an SSRI gain 4.2 kg more than non-users after a three-month treatment period [2], and that after 2.5 years of SSRI treatment, there is an increment of 2.5% of the initial body weight [3]. The risk factors associated with weight gain while receiving antidepressant medications are lower BMI at baseline, age under 65, and female gender [4]. However, the mechanisms of weight gain related to antidepressant use are not well known. Possible mechanisms include remission of major depression and increased neurotransmitters such as serotonin, which regulates feeding behaviors, energy expenditure [5, 6], and decreased brown adipose tissue thermogenesis [7].

Genetic variation is one of the factors that can alter a medication's efficacy by influencing its metabolism (i.e., pharmacokinetics), mechanism of action (i.e., pharmacodynamics), and even adverse side effects by gene-drug interactions. Because cytochrome P450 (CYP) enzymes contribute to phase I drug metabolism, CYP enzyme variation significantly impact treatment outcomes [8]. Pharmacogenomics offers the opportunity to optimize treatment considering these polymorphisms to develop a more personalized approach to antidepressant selection while reducing adverse drug events [9]. In 2013, the Clinical Pharmacogenetics Implementation Consortium (CPIC) developed dosing guidelines for paroxetine, citalopram, and sertraline based on their main metabolizer enzymes' phenotype status *CYP2C19* and/or *CYP2D6*. Different CYP enzymes are involved in SSRI metabolism; however, each drug has a dominant metabolizer enzyme. Thus, citalopram is mainly metabolized by *CYP2C19*, paroxetine by *CYP2D6*, fluoxetine by *CYP2D6* and *CYP2C9, and* sertraline by *CYP2D6* and *CYP2C19* [10]. The guidelines recommend a 50% reduction in the starting dose of citalopram, paroxetine, and sertraline in individuals with *CYP2D6* or *CYP2C19* poor metabolizer phenotype. In addition, the Food and Drug Administration has made recommendations for a maximum dosage of SSRIs in patients with specific metabolizer phenotypes [11]. There are currently little data detailing how CYP2D6 phenotypic status impacts the total amount of fluoxetine; hence, no gene-based dosage recommendations for fluoxetine have been provided.

Pharmacogenomics is a tool to personalize management in multiple areas, such as psychiatry and weight management [9, 12]. Multiple mood disorder studies have evaluated SSRI responsiveness for depression and lithium therapy in bipolar illness in GWAS studies and polygenic risk scores analysis [13–15]. These studies have found the link between genetic variants of obesity and SSRIs treatment response in depression [16]. However, a study investigating pharmacogenomics and weight gain in mood disorders is required. We hypothesized that patients with decreased metabolism of SSRIs by these cytochrome enzymes would be more likely to experience weight gain as a side effect. The study assesses the association between metabolizer phenotype and weight gain six months following SSRI prescription.

## Methods

### Study design

**Fig. 1:**
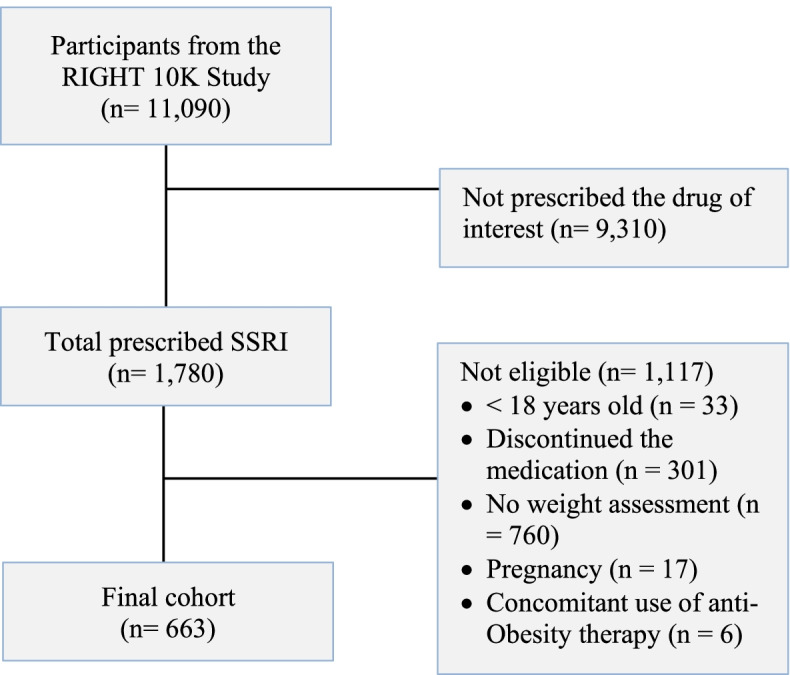
Study cohort

This retrospective cohort study approved by the Mayo Clinic Institutional Review Board (IRB 19–001,222) included participants from the Right Drug, Right Dose, Right Time (RIGHT) Study who underwent genetic sequencing of pharmacogenomic genes [17]. The RIGHT study included 11,090 participants, of which 60% were female and 97% were White. For the analyses, we considered participants from the RIGHT Study who had been prescribed citalopram, paroxetine, sertraline, or fluoxetine between 2004–2018. From those, we only included patients with a stable weight in the 6 months before starting the SSRI (*n* = 1,780). Of these, we exclude those who were < 18 years old, patients who did not have at least 6 months of treatment, did not have weight assessed during follow-up, had a history of bariatric surgery, were pregnant, or had a history of anti-obesity therapy (*n* = 1,117). The final analytic sample included 663 participants (Fig. 1). This study followed the Strengthening the Reporting of Observational Studies in Epidemiology (STROBE) reporting guideline (Additional file 2: Table S1).

### Data collection

Three physicians exhaustively reviewed the electronic medical record (EMR) to confirm inclusion and exclusion criteria. For the 663 participants included in the analysis, medication list, height, and body weight were extracted from the EMR, and comorbidities were extracted with ICD-9 and ICD-10 codes. Race was self-reported by study participants in the EMR, and race categories (American Indian or Alaska Native, Asian, Black or African American, Native Hawaiian or Other Pacific Islander, and White) were defined based on the US Office of Management and Budget’s Revisions to the Standards for the Classification of Federal Data on Race and Ethnicity. Body weight was extracted in kilograms at the time of prescription (± 2 weeks), three (± 2 weeks), and six (± 2 weeks) months of the initial prescription. Body mass index (BMI) was calculated with the formula weight in kilograms divided by height in meters squared.

### Exposure

As part of the RIGHT cohort, the Clinical Laboratory Improvement Amendments (CLIA)-certified and College of American Pathologists (CAP)-accredited Baylor College of Medicine's Human Genome Sequencing Center Clinical Laboratory sequenced 77 genes using version 3 (v.3.) of the PGRN-Seq assay (now termed PGx-seq) [18]. *CYP1A2, CYP2C9, CYP2C19, CYP2D6, CYP3A4, CYP3A5, DPYD, SLCO1B1, TPMT, UGT1A1, VKORC1, HLA-A,* and *HLA-B* were interpreted and reported by the Personalized Genomics Laboratory[17]. Genes studied are those pertinent to the enzymatic metabolism of the SSRI medications. The cytochrome P450 enzyme genes were *CYP2C19* for citalopram; *CYP2D6* for paroxetine; *CYP2D6* and *CYP2C9* for fluoxetine, and *CYP2C19* and *CYP2D6* sertraline [10]. Genotypes, including both rare and common variants, were translated into diplotypes using star allele nomenclature, when applicable, as described in PharmVar (www.pharmvar.org, last accessed 1/29/2022). Diplotypes were assigned a predicted metabolic phenotype (metabolizer status) using standard clinical laboratory processes, which rely on the assignment of the function of each allele present relative to a normal function (or “wild-type”) allele (Additional file 2: Table S2). Details of the genetic analysis have been previously reported [19]. Metabolizer status was classified as: poor/intermediate, normal (extensive), and rapid/ultra-rapid metabolizer [20]. We evaluate the concomitant use of inducers and inhibitors for P450-mediated metabolism (Additional file 2: Tables S3 and S4)[21].

### Outcome measurements

The outcome was calculated using the following formula:

$$TBWG\% = \frac{Body weight at 6 months - Baseline bodyweight }{Baseline bodyweight} \times 100$$

### Statistical analysis

Baseline anthropometric and demographics were not normally distributed according to the Shapiro-Wilks test and are summarized as median and interquartile range (IQR). Categorical data are presented as frequency and percentages. Categorical data were analyzed using Pearson's chi-squared test. ANCOVA models were used to assess the difference in total body weight change (%) with metabolizer status and BMI as a covariate. Multiple linear regression was calculated to evaluate the effect of metabolizer status and total body weight change (%) with normal metabolizer as a reference group and in three models, and parameter estimates with standard error (SE) were calculated for poor/intermediate metabolizer and rapid/ultra-rapid metabolizer. Model one included BMI; model two included BMI and age, and model three included BMI, sex, and age. The analysis also excluded patients with concomitant strong and moderate inducers and inhibitors for P450-mediated metabolism. To investigate whether the weight gain varies between BMI groups according to the World Health Organization classification (i.e., underweight and normal weight, overweight, and obesity), we stratified the analysis by normal weight, overweight, and obesity groups. ANCOVA models were used to assess the difference in total body weight change (%) with metabolizer status within these groups with BMI as a covariate, data are presented as mean and 95% confidence intervals (CI). Plots and statistical analyses were performed in SAS 9.2 (SAS Institute Inc., Cary, NC). *P*-values ≤ 0.05 were considered statistically significant.

## Results

The final cohort included 663 participants (age 61 [46 – 72] years, 76% females, and 94% white). The median BMI was 27.8 (24.0 – 32.9) kg/m^2^, 62% of our cohort had normal weight or overweight, and 38% had obesity (Table 1). The prevalence of the different metabolizer status phenotypes for *CYP2C19*, *CYP2D6,* and *CYP2C9* by SSRI is described in Table 2 and Additional file 3: Figure S1. The normal metabolizer phenotype was the most frequent in all cytochrome enzymes, except for *CYP2D6,* where the poor/intermediate metabolizer was the most predominant. The rapid/ultrarapid metabolizer phenotype was the least frequent. There were no significant differences in comorbidities among metabolizer phenotype (Additional file 2: Table S5).

**Table 1:** Participant characteristics in all participants and by drug. Data are shown as mean ± standard deviation or percentage

	**Total**	**Citalopram**	**Fluoxetine**	**Paroxetine**	**Sertraline**	*p*-value^*^
	*N* = 663	*N* = 202	*N* = 191	*N* = 107	*N* = 163	
**Demographics**						
Age, years	61 (46 – 72)	51 (40 – 66)	65 (53 – 76)	59 (48 – 69)	64 (46 – 77)	< 0.001
Gender, females	507 (76%)	158 (78%)	151 (79%)	80 (75%)	118 (72%)	0.44
Race, White	621 (94%)	190 (94%)	181 (95%)	100 (93%)	150 (92%)	0.79
**Anthropometrics**						
Weight, kg	78.0 (65.3 – 94)	76.2 (64.3 – 91.1)	82.0 (69.7 – 97)	74.2 (62 – 92.7)	76.7 (65 – 93.7)	0.03
BMI, kg/m^2^	27.8 (24.0 – 32.9)	27.2 (23.6 – 32.1)	30.0 (25.1 – 34.4)	26.3 (23.8 – 32.5)	27.7 (23.7 – 32.5)	0.009
**BMI Class**						
Class, underweight or normal weight	211 (32%)	61(30%)	46 (24%)	45 (35%)	59 (36%)	
Class, overweight	202 (30%)	79 (39%)	48 (25%)	25 (23%)	50 (31%)	
Class, obesity	250 (38%)	62 (31%)	97 (51%)	37 (42%)	52 (33%)	

Continuous data are summarized as median (IQR). Categorical data are presented as frequencies and percentages

Abbreviations used: *BMI* Body mass index

^*^p-value: calculated with ANOVA

**Table 2:** Distribution of phenotypes of cytochromes enzymes involved in the metabolism of citalopram, paroxetine, fluoxetine, and sertraline among the participants

	**Total**	**Citalopram**	**Fluoxetine**	**Paroxetine**	**Sertraline**
	*N* = 663	*N* = 202	*N* = 191	*N* = 107	*N* = 163
**CYP2C19**					
Poor/intermediate metabolizer, n	196 (30%)	58 (29%)			46 (28%)
Normal metabolizer, n	268 (40%)	83 (41%)			66 (41%)
Rapid/ultrarapid metabolizer, n	199 (30%)	61 (30%)			51 (31%)
**CYP2D6**					
Poor/intermediate metabolizer, n	462 (70%)		136 (71%)	70 (65%)	127 (78%)
Normal metabolizer, n	191 (29%)		53 (28.9%)	34 (32%)	31 (19%)
Rapid/ultrarapid metabolizer, n	10 (1%)		2 (0.1%)	3 (3%)	5 (3%)
**CYP2C9**					
Poor/intermediate metabolizer, n	234 (35%)		70 (37%)		
Normal metabolizer, n	429 (65%)		121 (63%)		

Categorical data are presented as frequencies and percentages

The detail medications used which inhibit or induce the CYP 450 enzymes by SSRI can be found in Additional file 2: Tables S3 and S4. From patients taking citalopram, 1 (0.5%) was concomitantly prescribed rifampin, a strong inducer for *CYP2C19*, and 3 (1.5%) were prescribed fluconazole or fluvoxamine, strong inhibitors for *CYP2C19*. From patients taking fluoxetine, 1 (0.05%) was concomitantly prescribed terbinafine, a strong inhibitor for *CYP2D6*, and 11 (5.7%) were prescribed a moderate inhibitor for *CYP2C9* or *CYP2D6* (i.e., fluconazole [*n* = 8], and duloxetine [*n* = 3]). From the patients taking paroxetine, 1 (1%) was prescribed duloxetine, a moderate inhibitor of *CYP2D6*. From patients taking sertraline, 1 (1%) was concomitantly prescribed phenytoin, a moderate inducer for *CYP2C19*, and 2 (1%) were prescribed fluconazole a strong inhibitor for *CYP2C19*.

### Total body weight gain % by metabolizer status

**Fig. 2:**
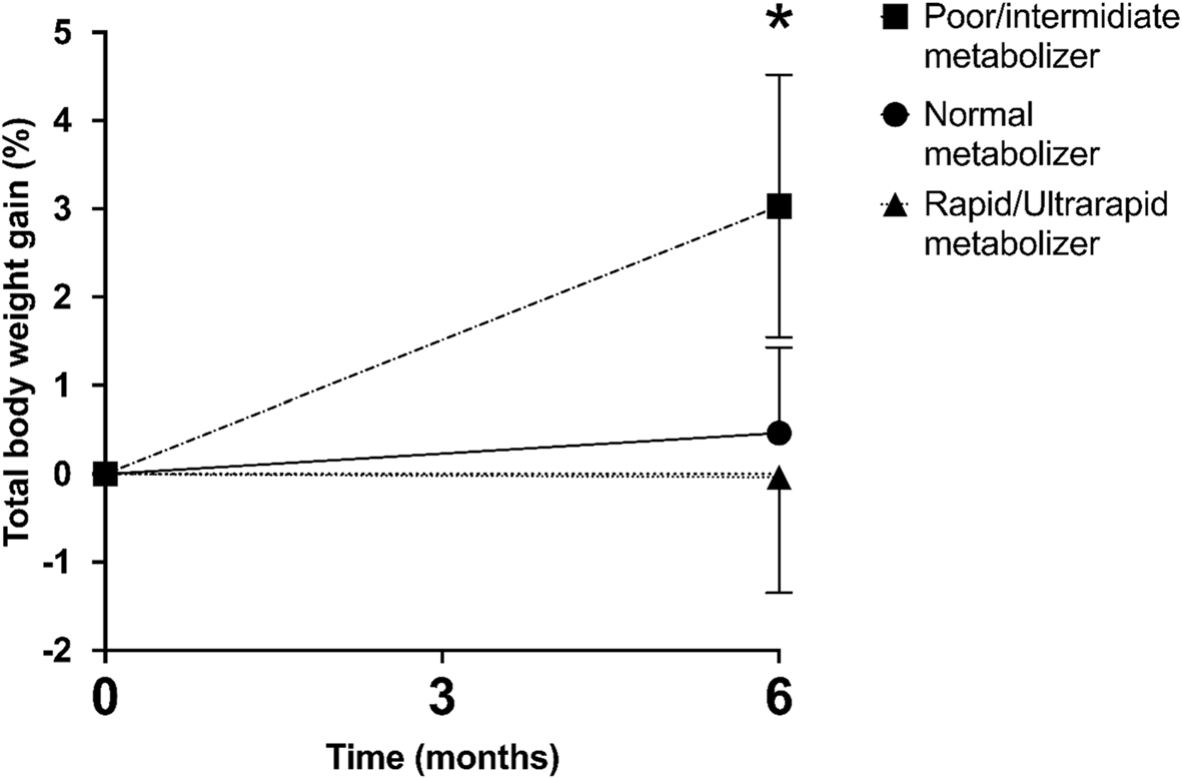
Effect of citalopram on total body weight by CYP2C19 phenotype. *p = 0.001

The total body weight gain percentage (TBWG %) at six months for the patients prescribed any SSRIs was 0.7% (-1.4 – 2.9). When analyzed by medication prescribed, TBWG % at six months for citalopram was 1.1% (-1.3 – 3.1), paroxetine 0.9% (-1.2 – 3.3), sertraline 1.1% (-1.0 – 2.9), and fluoxetine 0.1% (-1.9 – 2.6). For patients on citalopram, patients who were poor/intermediate *CYP2C19* metabolizers gained significantly more weight than normal and rapid/ultrarapid metabolizers (TBWG %: 2.6 [95% CI 1.3—4.1] vs. 0.4 [95% CI -0.5 – 1.3] vs. -0.1 [-95% CI -1.5—1.1], respectively; *p* = 0.001) (Fig. 2). After excluding 3 patients with an inducer or inhibitor of the CYP 450, patients who were poor/intermediate *CYP2C19* metabolizers gained significantly more weight than normal and rapid metabolizers (TBWG %: 2.2 [95% CI 1.1—4.0] vs. 0.4 [95% CI -0.5 – 1.5] vs. -0.2 [95% CI -1.3—1.4], respectively; *p* = 0.003). No significant difference was found in TBWG percentage at three or six months according to the *CYP2D6* phenotype for paroxetine, fluoxetine, and sertraline, *CYP2C9* for fluoxetine *CYP2C19* for fluoxetine and sertraline (Table 3).

**Table 3:** Total body weight gain percentage by CYP phenotype in participants prescribed with citalopram, paroxetine, fluoxetine, and sertraline

	**Poor/intermediate metabolizer**	**Normal metabolizer**	**Rapid/ultra-rapid metabolizer**	***p*- value**
**CYP2C19**				
**Citalopram**				
TBWG 6 months, %	2.6 (95% CI 1.3 – 4.1)	0.4 (95% CI -0.5 – 1.3)	-0.1 (95% CI -1.5 – 1.1)	**0.001**
**Sertraline**				
TBWG 6 months, %	0.9 (95% CI -0.09 – 2.1)	0.4 (95% CI -0.6 – 1.5)	1.7 (95% CI 0.7 – 2.9)	0.13
**CYP2D6**				
**Paroxetine**				
TBWG 6 months, %	0.7 (95% CI -0.1 – 1.5)	1.6 (95% CI 0.2 – 2.9)	0.7 (95% CI -4.8 – 6.1)	0.50
**Sertraline**				
TBWG 6 months, %	1.1 (95% CI 0.4 – 1.9)	0.8 (95% CI -0.3 – 1.8)	0.9 (95% CI -9.0 – 10.9)	0.98
**Fluoxetine**				
TBWG 6 months, %	0 (95% CI -0.7 – 0.7)	0.2 (95% CI -1.2 – 1.5)	1.3 (95% CI -3 – 3)	0.84
**CYP2C9**				
**Fluoxetine**				
TBWG 6 months, %	0.5 (95% CI -0.5 – 1.6)	-0.1 (95% CI -1.1 – 0.6)		0.33

Continuous data are summarized as mean and 95% confidence interval (CI)

Abbreviations used: *TBWG* Total Body Weight Gain

*p*-value: calculated with ANCOVA with metabolizer status and BMI as covariates

### Total body weight gain % by metabolizer status among BMI groups

Table 4 details the effect of metabolizer status and TBWG % after six months for each medication by BMI group. Patients in the overweight group that were prescribed citalopram who were poor/intermediate *CYP2C19* metabolizers gained significantly more weight than normal and rapid metabolizers (TBWG %: 3.0 [95% CI -0.3 – 6.4] vs. -0.3 [95% CI -2.1 – 1.5] vs. 1.3 [95% CI -3.6 – 1.1], respectively; p = 0.02). We did not observe any significant difference in TBWG % between metabolizer status among patients in the underweight, normal weight, or obesity group taking citalopram, fluoxetine, sertraline, or paroxetine (Table 5).

**Table 4:** Total body weight gain by obesity class and CYP phenotype in participants prescribed with citalopram, paroxetine, fluoxetine, and sertraline

**Normal and Underweight Group**								
**CYP2C19**								
		**Poor/intermediate metabolizer**		**Normal metabolizer**		**Rapid/ultra-rapid metabolizer**		***p***-**value**
Citalopram	TBWG 6 mths, %	*n* = 17	4.4 (95% CI 1.9 – 6.8)	*n* = 26	1.5 (95% CI -0.3 – 3.4)	*n* = 18	1.5 (95% CI -0.6 – 3.5)	0.11
Sertraline	TBWG 6 mths, %	*n* = 12	2.6 (95% CI 0.9 – 4.5)	*n* = 28	1.2 (95% CI -0.8 – 3.1)	*n* = 19	2.7 (95% CI 1.1 – 4.3)	0.32
**CYP2D6**								
		**Poor/intermediate metabolizer**		**Normal metabolizer**		**Rapid/ultra-rapid metabolizer**		***p*-value**
Paroxetine	TBWG 6 mths, %	*n* = 33	0.6 (95% CI -0.7 – 1.9)	*n* = 11	0.4 (95% CI -2.7 – 3.6)	*n* = 1	3.0	0.65
Sertraline	TBWG 6 mths, %	*n* = 49	1.8 (95% CI 0.6 – 3.1)	*n* = 8	1.4 (95% CI -0.5 – 3.4)	*n* = 2	6.7 (95% CI -55.1 – 69.6)	0.16
Fluoxetine	TBWG 6 mths, %	*n* = 32	1.2 (95% CI -0.3 – 2.7)	*n* = 14	0.9 (95% CI -1.8 – 3.6)			0.88
**CYP2C9**								
		**Poor/intermediate metabolizer**		**Normal metabolizer**		**Rapid/ultra-rapid metabolizer**		***p*-value**
Fluoxetine	TBWG 6 mths, %	*n* = 26	1.9 (95% CI -0.3 – 4.1)	*n* = 20	0.5 (95% CI -1.1 – 2.1)			0.60
**Overweight Group**								
**CYP2C19**								
		**Poor/intermediate metabolizer**		**Normal metabolizer**		**Rapid/ultra-rapid metabolizer**		***p*-value**
Citalopram	TBWG 6 mths, %	*n* = 21	3.0 (95% CI -0.3 – 6.4)	*n* = 34	-0.3 (95% CI -2.1 – 1.5)	*n* = 24	1.3 (95% CI -3.6 – 1.1)	**0.02**
Sertraline	TBWG 6 mths, %	*n* = 19	1.5 (95% CI -0.2 – 3.1)	*n* = 17	0.7 (95% CI -0.7 – 2.1)	*n* = 14	2.3 (95% CI 0.6 – 4.1)	0.44
**CYP2D6**								
		**Poor/intermediate metabolizer**		**Normal metabolizer**		**Rapid/ultra-rapid metabolizer**		***p*-value**
Paroxetine	TBWG 6 mths, %	*n* = 12	1.6 (95% CI -0.8 – 3.9)	*n* = 12	3.5 (95% CI 1.4 – 5.6)	*n* = 1	1.3	0.13
Sertraline	TBWG 6 mths, %	*n* = 40	1.3 (95% CI 0.3 – 2.3)	*n* = 8	2.3 (95% CI -0.5 – 3.4)	*n* = 2	0.9 (95% CI -25.0 – 26.8)	0.69
Fluoxetine	TBWG 6 mths, %	*n* = 28	1.2 (95% CI -2.9 – 0.4)	*n* = 20	0.5 (95% CI -2.2 – 3.2)			0.21
**CYP2C9**								
		**Poor/intermediate metabolizer**		**Normal metabolizer**		**Rapid/ultra-rapid metabolizer**		***p*-value**
Fluoxetine	TBWG 6 mths, %	*n* = 16	0.6 (95% CI -3.3 – 2.1)	*n* = 32	0.5 (95% CI -2.3 – 1.3)			0.91
**Obesity Group**								
**CYP2C19**								
		**Poor/intermediate metabolizer**		**Normal metabolizer**		**Rapid/ultra-rapid metabolizer**		***p*-value**
Citalopram	TBWG 6 mths, %	*n* = 20	0.8 (95% CI -0.5 – 1.9)	*n* = 23	0.4 (95% CI -0.8 – 1.7)	*n* = 19	-0.3 (95% CI -2.7 – 2.2)	0.18
Sertraline	TBWG 6 mths, %	*n* = 15	-0.9 (95% CI -3.1 – 1.2)	*n* = 21	-0.7 (95% CI -2.6 – 1.2)	*n* = 18	0.6 (95% CI -1.7 – 2.9)	0.52
**CYP2D6**								
		**Poor/intermediate metabolizer**		**Normal metabolizer**		**Rapid/ultra-rapid metabolizer**		***p*-value**
Paroxetine	TBWG 6 mths, %	*n* = 11	0.4 (95% CI -0.8 – 1.6)	*n* = 25	0.7 (95% CI -1.3 – 2.6)	*n* = 1	0.4	0.98
Sertraline	TBWG 6 mths, %	*n* = 38	0.1 (95% CI -1.6 – 1.3)	*n* = 15	0.3 (95% CI -1.8 – 1.2)	*n* = 1	-10.6	0.26
Fluoxetine	TBWG 6 mths, %	*n* = 19	0.1 (95% CI -0.9 – 1.0)	*n* = 76	-0.9 (95% CI -3.1 – 1.2)	*n* = 2	1.3 (95% CI -33.0 – 35.6)	0.79
**CYP2C9**								
		**Poor/intermediate metabolizer**		**Normal metabolizer**		**Rapid/ultra-rapid metabolizer**		***p*-value**
Fluoxetine	TBWG 6 mths, %	*n* = 34	0.2 (95% CI -1.1 – 1.5)	*n* = 63	-0.3 (95% CI -1.5 – 0.8)			0.30

Continuous data are summarized as mean (95% CI)

Abbreviations used: *CI* confidence interval, *TBWG* Total Body Weight Gain

*p*-value: calculated with ANCOVA with metabolizer status and BMI as covariate

**Table 5:** Multiple regression variate analysis. Total body weight gain by obesity class and CYP phenotype in participants prescribed with citalopram, paroxetine, fluoxetine, and sertraline

**CYP2C19**													
		**Model 1**				**Model 2**				**Model 3**			
		**Poor/intermediate metabolizer**		**Rapid/ultra-rapid metabolizer**		**Poor/intermediate metabolizer**		**Rapid/ultra-rapid metabolizer**		**Poor/intermediate metabolizer**		**Rapid/ultra-rapid metabolizer**	
		**PE**	*p*-value^*^	**PE**	*p*-value^*^	**PE**	***p*-value**^**+**^	**PE**	**p-value**^**+**^	**PE**	***p*-value**^**++**^	**PE**	***p*-value**^**++**^
Citalopram	TBWG 6 mths, %	1.7 (0.5)	**0.001**	-1.2 (0.5)	**0.01**	1.7 (0.6)	**0.001**	-1.2 (0.5)	**0.02**	1.7 (0.5)	**0.001**	-1.2 (0.5)	**0.02**
Sertraline	TBWG 6 mths, %	-0.03 (0.4)	0.94	0.7 (0.4)	0.08	0.01 (0.4)	0.99	0.7 (0.4)	0.10	0.01 (0.4)	0.99	0.7 (0.4)	0.11
**CYP2D6**													
		**Model 1**				**Model 2**				**Model 3**			
		**Poor/intermediate metabolizer**		**Rapid/ultra-rapid metabolizer**		**Poor/intermediate metabolizer**		**Rapid/ultra-rapid metabolizer**		**Poor/intermediate metabolizer**		**Rapid/ultra-rapid metabolizer**	
		**PE**	*p*-value^*^	**PE**	*p*-value^*^	**PE**	***p*-value**^**+**^	**PE**	***p*-value**^**+**^	**PE**	**p-value**^**++**^	**PE**	**p-value**^**++**^
Paroxetine	TBWG 6 mths, %	-0.3 (1.4)	0.82	-0.3 (0.7)	0.71	-0.4 (0.7)	0.61	-0.1 (1.4)	0.96	0.4 (0.7)	0.61	-0.1 (1.4)	0.96
Sertraline	TBWG 6 mths, %	0.1 (0.6)	0.76	-0.3 (1.2)	0.80	0.1 (0.6)	0.89	-0.3 (1.2)	0.77	0.1 (0.6)	0.91	0.3 (1.2)	0.77
Fluoxetine	TBWG 6 mths, %	-0.6 (1.1)	0.58	1.2 (2.2)	0.57	-0.6 (1.1)	0.57	1.3 (2.2)	0.50	-0.6 (1.1)	0.60	1.2 (2.2)	0.57
**CYP2C9**													
		**Model 1**				**Model 2**				**Model 3**			
		**Poor/intermediate metabolizer**		**Rapid/ultra-rapid metabolizer**		**Poor/intermediate metabolizer**		**Rapid/ultra-rapid metabolizer**		**Poor/intermediate metabolizer**		**Rapid/ultra-rapid metabolizer**	
		**PE**	*p*-value^*^	**PE**	*p*-value^*^	**PE**	***p*-value**^**+**^	**PE**	***p*-value**^**+**^	**PE**	***p*-value**^**++**^	**PE**	***p*-value**^**++**^
Fluoxetine	TBWG 6 mths, %	0.3 (0.3)	0.33			0.3 (0.6)	0.33			0.3 (0.3)	0.39		

*p*-value: multiveriate linear regression with normal metabolizar as reference group

^*^ model including BMI

^+^ model including BMI and age

^++^ model including BMI, age and sex

Abbreviations used: *BMI* body mass index; *PE* parameter estimates, *TBWG* Total Body Weight Gain

### Effect of metabolizer status on total body weight gain %

Multiple linear regression was calculated to evaluate the effect of metabolizer status and TBWG % after six months for each medication. For citalopram, when adjusting for BMI, poor/intermediate *CYP2C19* metabolizer status resulted in a weight gain of 1.7% (Standard Error [SE] 0.5; *p* = 0.001), while rapid/ultrarapid metabolizer status resulted in a decrease of 1.2% (SE 0.5; *p* = 0.01). This effect remained significant after adjusting for BMI and age where poor/intermediate *CYP2C19* metabolizer status resulted in a weight gain of 1.7% (Standard Error [SE] 0.6; *p* = 0.001), while rapid/ultrarapid metabolizer status resulted in a decrease of 1.2% (SE 0.5; *p* = 0.02). This trend was also seen after adjusting for BMI, sex, and age where poor/intermediate *CYP2C19* metabolizer status resulted in a weight gain of 1.7% (Standard Error [SE] 0.5; *p* = 0.001), while rapid/ultrarapid metabolizer status resulted in a decrease of 1.2% (SE 0.5; *p* = 0.02). No significant effect was found for *CYP2D6* phenotype for paroxetine, fluoxetine, and sertraline, *CYP2C9* for fluoxetine, *CYP2C19* for fluoxetine, and sertraline when adjusting for BMI, BMI and age, or BMI, age, and sex.

## Discussion

The current study identified that poor/intermediate metabolizer status for *CYP2C19* is associated with a 1.7% more weight gain after 6 months than normal metabolizers in patients taking citalopram. This study shows that this remains significant among patients with overweight, where patients with poor/intermediate metabolizer status for *CYP2C19* and taking citalopram had a TBWG of 3.0%. There were no disparities in comorbidities across individuals with various metabolizer statuses, and the difference in weight change remained after excluding patients who were using a CYP 450 inducer or inhibitor concurrently, highlighting the importance of the metabolizer status. Our results are generally consistent with previous studies showing the effect of citalopram on body weight [4, 22]. Aldrich et al. conducted a retrospective study using an electronic medical record of 263 youth with anxiety and depression prescribed citalopram. They showed a significant association between poor *CYP2C19* metabolizer phenotype and earlier weight gain after 45 days of treatment [23]. The weight changes related to other antidepressants were not connected with the other pharmacogenomic genes of interest.

Our findings are also consistent with previous studies in which fluoxetine and sertraline have shown minimal effects on weight gain [24]. Conversely, paroxetine has demonstrated a greater risk of weight gain. Serretti et al. reported that the mean weight difference during 8 months of treatment was 2.73 kg for paroxetine [25]. These findings were not replicated in our cohort treated with paroxetine; this discrepancy might be explained by the different intervals of the observations in the two studies.

A number of reasons complicate weight changes in individuals receiving depression medication; they may indicate an improvement in those who have lost weight due to their depression, but they can also be a side effect of the treatment. In our study, weight gain was seen in overweight patients on citalopram with poor/intermediate *CYP2C19* metabolizer status. Previous research has found that participants who considered their weight status as overweight were more likely to gain weight in the future [26]. We found that weight increase in patients treated with citalopram was significant even after controlling for BMI, indicating the importance of metabolizer status. As a result, it is critical to identify individuals who are prone to weight gain and risk factors that may contribute to it. It is crucial to underline that a decision tool such as pharmacogenomics may be more effective in these individuals as an ad hoc instrument.

Multiple drugs with indications for chronic weight management have been authorized with improved safety profiles [27]. However, in a patient-centered care model, it is important to recognize barriers that may decrease to less effective and efficient weight management and that negatively impact weight loss outcomes. One of these barriers is weight gain as a medications’ side effect, i.e. obesogenic drugs [28]. According to the findings of a patient survey, the most common reason for discontinuing antidepressant medication is a lack of effectiveness. However, up to 27% of patients who reported noncompliance discontinued the drug due to weight increase [29]. Previous research has looked at the link between metabolizer status and medicine discontinuation; however, no convincing relationship has been identified due to study design and sample collection [30]. More research is needed to determine the true impact of metabolizer status on drug discontinuation, particularly in individuals who are overweight and using SSRIs.

Despite their widespread usage of antidepressant medicine, initial drug selection success might be lower. According to the findings of the Sequenced Treatment Alternatives to Relieve Depression (STAR*D) trial, only one-third of the patients achieved remission within the first treatment level [31]. Previous studies have found the clinical benefits of using pharmacogenomics to tailor the therapeutic approach in patients with major depressive disorder and anxiety [32–37]. Poor metabolizers taking escitalopram resulted in a greater rate of therapeutic failure, indicating the potential clinical value of *CYP2C19* genotyping for individualization of escitalopram [38]. However, there has not been any difference in adverse drug events reported between pharmacogenomic tailored treatment compared with controls [8, 9, 32].

Previous studies have retrospectively assessed side effects and metabolizer status with antidepressants such as tricyclic antidepressants (TCAs), serotonin-noradrenaline reuptake inhibitors (SNRIs), and SSRIs [39, 40]. In terms of side effects and *CYP2C19* and citalopram/escitalopram, a meta-analysis of 2037 patients found that, compared to normal metabolizers, *CYP2C19* poor metabolizers had a greater risk of gastrointestinal, neurological, and sexual adverse effects [41]. The Patient Rated Inventory of Side Effects (PRISE), which includes weight gain among other items and covers 9 categories and 32 items, is a typical measure used to evaluate side effects in most pharmacogenomics studies. Most research, however, focuses on side effects associated with a specific organ or system, which may restrict the relationship between pharmacogenomic studies with weight gain. Another study has looked at variations in the genes *CYP2D6*, *CYP2C19*, and *CYP2C9* in patients taking SSRIs and tolerability and did not find a clear pharmacogenetic explanation for side effects [30]. In a recent study of 9500 participants, poor metabolizers were at higher risk of side effects adding to the evidence for a link between *CYP2C19* metabolism and SSRI tolerability [42]. However, tolerability and side effects were evaluated by a survey using a qualitative assessment considering weight gain. The relationship between poor *CYP2C19* metabolizer status and early weight gain documented in the medical record in children using escitalopram or citalopram has been described, adding to the data connecting metabolizer status and weight increase [43]. This is the first study to objectively evaluate one of the common side effects of SSRIs, regardless of the response to treatment in adults.

Although studies on the association between citalopram blood concentrations and pharmaceutical effectiveness and tolerability are lacking, it is usually assumed that a 50% difference in blood concentration will have a clinical impact [10, 44]. Because of the increase in concentration for *CYP2C19* poor metabolizers compared to normal metabolizers, there is an implied risk of adverse outcomes, and the recommendation is to reduce the citalopram dose by 50% [10]. Further studies are needed to evaluate drug blood concentrations and weight gain according to metabolizer status.

Our study has some limitations. First, the low prevalence of some CYP enzyme phenotypes (*CYP2D6* rapid/ultrarapid metabolizer) and the small sample size might cause a type II error in assessing our primary outcome. This limits the ability to detect a difference in TBWG % with other enzyme phenotypes. Importantly, given the nature of the study, we could not include all patients prescribed an SSRI because a few had no follow up at our institution. Second, the generalizability of the data is limited by the retrospective nature of our study and does not establish a causal link between CYP enzyme phenotypes and weight gain. Third, it is difficult to investigate characteristics such as drug compliance since medical record data varies so much between health care providers, and the influence of polypharmacy on patient outcomes was not evaluated. This is an important aspect to consider in future research. Previously, tricyclic antidepressants, which have a more profound effect on bodyweight, weight gain, have been a clear limitation of compliance to the medication. Weight gain in our cohort may have resulted in noncompliance, concealing the differences in weight in our cohort among drugs that have previously been associated with higher weight gain. Fourth, there are ascertainment biases inherent to a study conducted in tertiary care centers with a study population that is predominately White. Fifth, the mood response to the SSRI was not formally recorded with validated questionnaires, and this outcome was unclear from retrospective chart review during data gathering. Thus, response to treatment of depression could confound weight gain or weight loss.

In addition to the CYP genes, other genes related to the serotonin and norepinephrine signaling have been implicated with the therapeutic responses to SSRI. Previous studies have evaluated the effect of genetic variants on change in depressive symptoms and found significant associations with several variants in the serotonin receptor gene (*HTR2A*) and the response to escitalopram, the norepinephrine transporter gene (*SLC6A2*) and the response to nortriptyline, and the glucocorticoid receptor gene (*NR3C1*) and the response to both nortriptyline and escitalopram [45]. Here, we did not evaluate the effect of genetic variants that may affect the response to drugs with different mechanisms of action. Studies have also examined genes related to weight gain and SSRIs, such as catechol-O-methyltransferase (*COMT),* tryptophan hydroxylase 1 (*TPH1), HTR2C,* and serotonin transporter gene *(SLC6A4).* The evidence shows that GG *COMT* and AA *TPH1* genotypes have more weight gain outcomes than *HTR2C* and *SLC6A4* polymorphism[9, 16, 37]. Our research did not cover the effect of other genes involved in the SSRI metabolisms; therefore, further research focusing on other enzymes involved in the SSRI metabolism is needed to understand the variability in weight gain response to this class of medication. The study’s strengths include a high level of detail regarding CYP genotypes polymorphisms and weight loss outcomes and complications after bariatric surgery. It is, to our knowledge, the largest research evaluating weight change outcomes, combining the administration of CYP inducers and inhibitors at the same time.

## Conclusions

In conclusion, we have performed a retrospective pharmacogenomics study to understand the SSRIs’ common weight gain side effects. We showed that the *CYP2C19* genotype might explain weight gain in citalopram patients, and it might become a projection tool for preventing weight gain and obesity, particularly in patients who are overweight. Further studies are needed to validate this observation in prospective trials.

## Supplementary Information

**Additional file 1:** 

**Additional file 2: Table S1. **STROBE reporting check list. T**able S2.** Variants/star alleles considered in this study. **Table S3.** Clinical inducers and inhibitors for P450-mediated metabolism considered. [46]. **Table S4.** Distribution of concomitant use of clinical inducers and inhibitors for P450-mediated metabolism by antidepressant. **Table S5.** Distribution of comorbidities by metabolizer status. CYP2C9>

**Additional file 3: Figure S1. **Distribution of phenotypes of cytochromes enzymes involved in the metabolism of citalopram, paroxetine, fluoxetine, and sertraline among the participants.

## Data Availability

The raw data supporting the conclusions of this manuscript will be made available by the authors, without undue reservation, to any qualified researcher.

## Abbreviations

BMI: Body mass index
CPIC: Clinical Pharmacogenetics Implementation Consortium
CYP: Cytochrome P450
EMR: Electronic medical record
IQR: Interquartile range
PE: Parameter estimates
RIGHT: Right Drug, Right Dose, Right Time
SSRIs: Selective serotonin reuptake inhibitors
TBWG: Total Body Weight Gain
