# Supplementary material for: Association between CYP metabolizer phenotypes and selective serotonin reuptake inhibitors induced weight gain: a retrospective cohort study
Source: BMC Med. 2022 Jul 26;20:261. doi: 10.1186/s12916-022-02433-x (PMC9317126; doi:10.1186/s12916-022-02433-x)
Supplement: Supplementary file 1 — Additional file 1: [file 12916_2022_2433_MOESM1_ESM.docx]

**Additional file 1:**

**Methods:**

Co-morbidities were extracted using ICD-9 and ICD-10 codes as follows: ICD-9: Congestive heart failure (i.e., 398.91, 402.01, 402.11, 402.91, 404.01, 404.03, 404.11, 404.13, 404.91, 404.93, 425.4 - 425.9, 428.x), diabetes (i.e., 250.0 - 250.3, 250.8, 250.9, 250.4 - 250.7), renal disease (i.e., 403.01, 403.11, 403.91, 404.02, 404.03, 404.12, 404.13, 404.92, 404.93, 582.x, 583.0 - 583.7, 585.x, 586.x, 588.0, V42.0, V45.1, V56.x), and any malignancy, including lymphoma and leukemia (i.e., 140.x - 172.x, 174.x - 195.8, 200.x - 208.x, 238.6). ICD-10: Congestive heart failure (i.e., I09.9, I11.0, I13.0, I13.2, I25.5, I42.0, I42.5 - I42.9, I43.x, I50.x, P29.0), diabetes (i.e., E10.0, E10.1, E10.6, E10.8, E10.9, E11.0, E11.1, E11.6, E11.8, E11.9, E12.0, E12.1, E12.6, E12.8, E12.9, E13.0, E13.1, E13.6, E13.8, E13.9, E14.0, E14.1, E14.6, E14.8, E14.9, E10.2 - E10.5, E10.7, E11.2 - E11.5, E11.7, E12.2 - E12.5, E12.7, E13.2 - E13.5, E13.7, E14.2 - E14.5, E14.7), renal disease (i.e., I12.0, I13.1, N03.2 - N03.7, N05.2 - N05.7, N18.x, N19.x, N25.0, Z49.0 - Z49.2, Z94.0, Z99.2) and any malignancy, including lymphoma and leukaemia (i.e., C00.x - C26.x, C30.x - C34.x, C37.x - C41.x, C43.x, C45.x - C58.x, C60.x - C76.x, C81.x - C85.x, C88.x, C90.x - C97.x)
