# Supplementary material for: Association between CYP metabolizer phenotypes and selective serotonin reuptake inhibitors induced weight gain: a retrospective cohort study
Source: BMC Med. 2022 Jul 26;20:261. doi: 10.1186/s12916-022-02433-x (PMC9317126; doi:10.1186/s12916-022-02433-x)
Supplement: Supplementary file 2 — Additional file 2: Table S1. STROBE reporting check list. Table S2. Variants/star alleles considered in this study. Table S3. Clinical inducers and inhibitors for P450-mediated metabolism considered. [46]. Table S4. Distribution of concomitant use of clinical inducers and inhibitors for P450-mediated metabolism by antidepressant. Table S5. Distribution of comorbidities by metabolizer status. CYP2C9> [file 12916_2022_2433_MOESM2_ESM.docx]

**Additional file 2:** **Tables 1-5**

**Table S1**. STROBE reporting check list

|  | Item No | Recommendation | Page No |
| --- | --- | --- | --- |
| **Title and abstract** | 1 | (*a*) Indicate the study’s design with a commonly used term in the title or the abstract | 1 |
|  |  | (*b*) Provide in the abstract an informative and balanced summary of what was done and what was found | 2 |
| Introduction | | | |
| Background/rationale | 2 | Explain the scientific background and rationale for the investigation being reported | 4-5 |
| Objectives | 3 | State specific objectives, including any prespecified hypotheses | 5 |
| Methods | | | |
| Study design | 4 | Present key elements of study design early in the paper | 5-6 |
| Setting | 5 | Describe the setting, locations, and relevant dates, including periods of recruitment, exposure, follow-up, and data collection | 6 |
| Participants | 6 | (*a*) Give the eligibility criteria, and the sources and methods of case ascertainment and control selection. Give the rationale for the choice of cases and controls | 6, fig 1 |
|  |  | (*b*) For matched studies, give matching criteria and the number of controls per case | NA |
| Variables | 7 | Clearly define all outcomes, exposures, predictors, potential confounders, and effect modifiers. Give diagnostic criteria, if applicable | 6-7 |
| Data sources/ measurement | 8* | For each variable of interest, give sources of data and details of methods of assessment (measurement). Describe comparability of assessment methods if there is more than one group | 7, supplement |
| Bias | 9 | Describe any efforts to address potential sources of bias | 7 |
| Study size | 10 | Explain how the study size was arrived at | NA |
| Quantitative variables | 11 | Explain how quantitative variables were handled in the analyses. If applicable, describe which groupings were chosen and why | 7 |
| Statistical methods | 12 | (*a*) Describe all statistical methods, including those used to control for confounding | 7 |
|  |  | (*b*) Describe any methods used to examine subgroups and interactions | NA |
|  |  | (*c*) Explain how missing data were addressed | 7 |
|  |  | (*d*) If applicable, explain how matching of cases and controls was addressed | NA |
|  |  | (*e*) Describe any sensitivity analyses | NA |
| Results | | | |
| Participants | 13* | (a) Report numbers of individuals at each stage of study—eg numbers potentially eligible, examined for eligibility, confirmed eligible, included in the study, completing follow-up, and analyzed | 6, Fig 1 |
|  |  | (b) Give reasons for non-participation at each stage | 6, Fig 1 |
|  |  | (c) Consider use of a flow diagram | Fig 1 |
| Descriptive data | 14* | (a) Give characteristics of study participants (eg demographic, clinical, social) and information on exposures and potential confounders | 7, Table 1 |
|  |  | (b) Indicate number of participants with missing data for each variable of interest | 6 |
| Outcome data | 15* | Report numbers in each exposure category, or summary measures of exposure | 7-8, Table 1-2 |
| Main results | 16 | (*a*) Give unadjusted estimates and, if applicable, confounder-adjusted estimates and their precision (eg, 95% confidence interval). Make clear which confounders were adjusted for and why they were included | 6, Table 3,4 and 5 |
|  |  | (*b*) Report category boundaries when continuous variables were categorized | 6, tables |
|  |  | (*c*) If relevant, consider translating estimates of relative risk into absolute risk for a meaningful time period | NA |
| Other analyses | 17 | Report other analyses done—eg analyses of subgroups and interactions, and sensitivity analyses | NA |
| **Discussion** |  |  |  |
| Key results | 18 | Summarize key results with reference to study objectives | 9-10 |
| Limitations | 19 | Discuss limitations of the study, taking into account sources of potential bias or imprecision. Discuss both direction and magnitude of any potential bias | 11 |
| Interpretation | 20 | Give a cautious overall interpretation of results considering objectives, limitations, multiplicity of analyses, results from similar studies, and other relevant evidence | 11 |
| Generalizability | 21 | Discuss the generalizability (external validity) of the study results | 11 |
| **Other information** |  |  |  |
| Funding | 22 | Give the source of funding and the role of the funders for the present study and, if applicable, for the original study on which the present article is based | 1 |

**Table S2**. Variants/star alleles considered in this study.

| **Phenotype** | **Definition** | ***CYP2D6*** | ***CYP2C19*** | ***CYP2C9*** |
| --- | --- | --- | --- | --- |
| **Poor Metabolizer/ Intermediate Metabolizer** | Poor: No function and/or decreased function alleles  Intermediate: Combinations of normal function, decreased function, and/or non-functional alleles | ****3/*3, *3/*4, *3/*5, *3/*68+*4, *4/*4, *4/*4N+*4, *4/*4x2, *4/*5, *4/*6, *4/*68+*4, *4N+*4/*68+*4, *5/*5, *68+*4/*5, *68+*4/*68+*4, *1/*3, *1/*4, *1/*4N+*4, *1/*4x2, *1/*5, *1/*6, *1/*13, *1/*68+*4, *1/*68xN+*4, *10/*36+*10, *10/*41, *13+*1/*4, *13+*2A/*4, *2Ax2/*68+*4, *2A/*3, *2A/*4, *2A/*4N+*4, *2A/*4x2, *2A/*5, *2A/*6, *2A/*68+*4, *2A/*13, *3/*35, *4/*35, *4x2/*35, *6/*33, *6/*35, *68+*4/*35, *9/*41, *1/*10, *1/*17, *1/*28, *1/*29, *1/*36+*10, *1/*41, *1/*59, *1/*9, *10/*35, *2/*45, *28/*35, *2A/*10, *2A/*22, *2A/*28, *2A/*2A, *2A/*41, *2A/*9, *13/*32/*2/*5, *2A/*6, *3/*10, *3/*41, *4/*9, *4/*10, *4/*41, *5/*9, *5/*22, *5/*41, *6/*9, *6/*10, *6/*41, *68+4/*10, *68+*4/*41, *68+*4/*9, *68x2+*4/*41, *32/*35, *33/*41, *35/*41, *9/*33, *9/35, *10/*28, *22/*41, *28/*41*** | ****2/*2, *2/*4, *1/*2, *1/*4, *1/*6, *1/*8, *1/*9, *1/*10, *2/*17*** | ****2/*3, 3/*3, *3/*44, *1/*2, *1, *3, *1/*8, *1/*11, *1/*45, *2/*2*** |
| **Normal Metabolizer** | Normal function alleles, and some decreased function alleles | ****1/*1, *1/*2A, *1/*33, *1/*35, *1x2/*4, *1x2/*4N+*4, *1x2/*68+*4, *2A/*33, *2A/*35, *33/*35, *35/*35*** | ****1/*1*** | ****1/*1, *1/*12*** |
| **Rapid Metabolizer/ Ultrarapid Metabolizer** | Rapid: Increased and normal function alleles  Ultrarapid: Increased function alleles and additional copies of normal alleles | ****1/*2Ax2, *1x2/*2A, *1x2/*41, *1/*1x2, *1x2/*33*** | ****1/*17, *17/*17*** | ***N/A*** |

**Table S3**. Clinical inducers and inhibitors for P450-mediated metabolism considered. (1)

|  | **Strong Inducers** | **Moderate Inducers** | **Weak  Inducers** |
| --- | --- | --- | --- |
| **CYP2C9** |  | Enzalutamide (n=0), rifampin (n=0) | apalutamide (n=0), aprepitant (n=0), carbamazepine (n=2), ritonavir (n=0) |
| **CYP2C19** | rifampin (n =1) | apalutamide (n=0), efavirenz (n=0), enzalutamide (n=0), phenytoin (n=1) | ritonavir (n=0) |
|  | | | |
|  | **Strong inhibitors** | **Moderate inhibitors** | **Weak inhibitors** |
| **CYP2C9** |  | amiodarone (n=1), fluconazole (n=13), miconazole (n=0), piperine (n=0) | diosmin (n=0), disulfiram (n=0) fluvastatin (n=0) fluvoxamine (n=0) voriconazole (n=0) |
| **CYP2C19** | fluconazole (n=13)  fluvoxamine (n=1) ticlopidine ((n=0) | felbamate (n=0) | omeprazole (n=75)  voriconazole (n=0) |
| **CYP2D6** | bupropion (n=0)  quinidine (n=0)  terbinafine (n=1) | abiraterone (n=0)  cinacalcet (n=0) duloxetine (n=4) lorcaserin (n=0) mirabegron (n=0) | amiodarone (n=2) celecoxib (n=21) cimetidine (n=0) clobazam (n=0)  cobicistat (n=0) fluvoxamine (n=0) labetalol (n=9)  ritonavir (n=0) vemurafenib (n=0) |

**Table S4.** Distribution of concomitant use of clinical inducers and inhibitors for P450-mediated metabolism by antidepressant.

|  | **Citalopram** | **Fluoxetine** | **Paroxetine** | **Sertraline** | **Total** |
| --- | --- | --- | --- | --- | --- |
|  | N = 202 | N = 191 | N = 107 | N = 163 | N = 663 |
| **Inducers** | | | | | |
| CYP2C19: Rifampin | 1* | 0 | 0 | 0 | 1 |
| CYP2C19 Phenytoin | 0 | 0 | 0 | 1* | 1 |
| CYP2C9: Carbamazepine | 2 | 0 | 0 | 0 | 2 |
| **Inhibitors** | | | | | |
| CYP2C19/ CYP2C9: Fluconazole | 2* | 8* | 1 | 2* | 13 |
| CYP2C19: Fluvoxamine | 1* | 0 | 0 | 0 | 1 |
| CYP2C19: Omeprazole | 25* | 27 | 9 | 14* | 75 |
| CYP2C9: Amiodarone | 1 | 0 | 0 | 0 | 1 |
| CYP2D6: Celecoxib | 6 | 6* | 5* | 4* | 21 |
| CYP2D6: Labetalol | 4 | 2 | 1* | 2* | 9 |
| CYP2D6: Terbinafine | 1 | 1* | 0 | 0 | 2 |
| CYP2D6: Duloxetine | 0 | 3* | 1* | 0 | 4 |
| Significant interactions between medication and CYP are marked with * | | | | | |

**Table S5**. Distribution of comorbidities by metabolizer status.

| **CYP2C9** | | | | |
| --- | --- | --- | --- | --- |
|  | Poor/ intermediate metabolizer  (n = 234) | Normal metabolizer (n = 429) |  | p-value |
| CHF | 36 (8%) | 27 (12%) |  | 0.18 |
| Diabetes | 106 (25%) | 50 (21%) |  | 0.33 |
| Kidney Disease | 60 (14%) | 37 (16%) |  | 0.52 |
| Neoplasm | 156 (36%) | 80 (34%) |  | 0.58 |
| **CYP2C19** | | | | |
|  | Poor/ intermediate metabolizer  (n = 196) | Normal metabolizer (n = 268) | Rapid/ultrarapid metabolizer (n = 199) | p-value |
| CHF | 19 (10%) | 31 (12%) | 13 (7%) | 0.18 |
| Diabetes | 49 (25%) | 62 (23%) | 45 (23%) | 0.83 |
| Kidney Disease | 23 (12%) | 44 (16%) | 30 (15%) | 0.36 |
| Neoplasm | 75 (38%) | 95 (35%) | 66 (33%) | 0.57 |
| **CYP2D6** | | | | |
|  | Poor/ intermediate metabolizer  (n = 462) | Normal metabolizer (n = 191) | Rapid/ultrarapid metabolizer (n = 10) | p-value |
| CHF | 46 (10%) | 16 (8%) | 1 (10%) | 0.82 |
| Diabetes | 112 (24%) | 42 (22%) | 2 (20%) | 0.79 |
| Kidney Disease | 66 (14%) | 31 (16%) | 0 (0%) | 0.34 |
| Neoplasm | 158 (14%) | 76 (40%) | 1 (10%) | 0.22 |
| Abbreviations: CHF, cardiac heart failure.  p-value: Pearson's chi-squared test | | | | |
