# Supplementary material for: Association between CYP metabolizer phenotypes and selective serotonin reuptake inhibitors induced weight gain: a retrospective cohort study
Source: BMC Med. 2022 Jul 26;20:261. doi: 10.1186/s12916-022-02433-x (PMC9317126; doi:10.1186/s12916-022-02433-x)
Supplement: Supplementary file 3 — Additional file 3: Figure S1. Distribution of phenotypes of cytochromes enzymes involved in the metabolism of citalopram, paroxetine, fluoxetine, and sertraline among the participants. [file 12916_2022_2433_MOESM3_ESM.docx]

**Additional file 3:** **Figure S1.** Distribution of phenotypes of cytochromes enzymes involved in the metabolism of citalopram, paroxetine, fluoxetine, and sertraline among the participants.

**
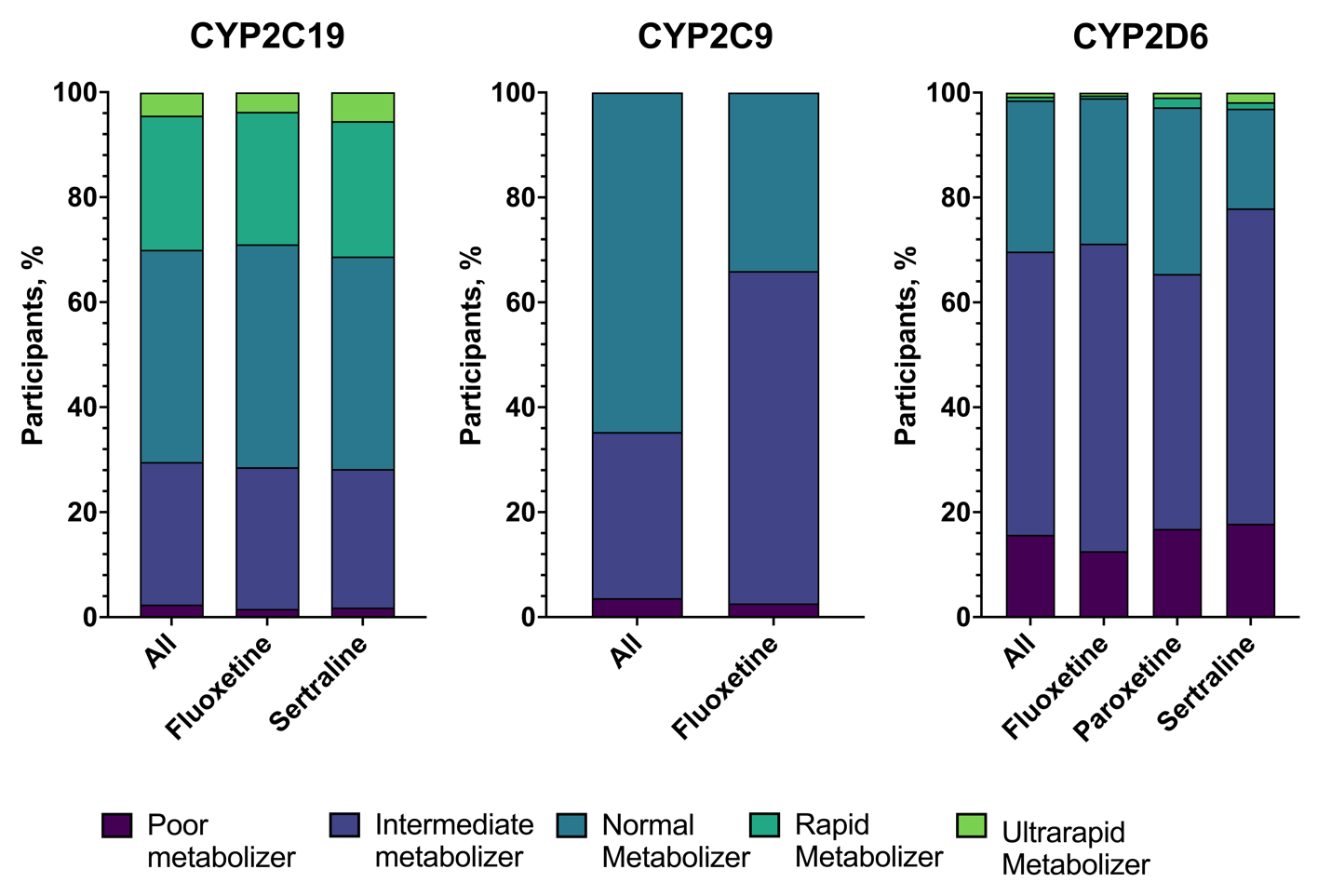
**
